# Correction: Polyterrylenes: synthesis and regioregularity effect on p-type charge transport and deep-red light photodetection in OFETs

**DOI:** 10.1039/d6sc90068b

**Published:** 2026-03-24

**Authors:** Chitrak Ghosh, Minji Chung, Hayeong Park, Aniket Jitendra Talreja, Ullrich Scherf, Joon Hak Oh, Suman Kalyan Samanta

**Affiliations:** a Department of Chemistry, Indian Institute of Technology Kharagpur Kharagpur 721302 India sksamanta@chem.iitkgp.ac.in; b School of Chemical and Biological Engineering, Institute of Chemical Processes, Seoul National University Seoul 08826 Republic of Korea joonhoh@snu.ac.kr; c Macromolecular Chemistry Group (buwmakro), Wuppertal Center for Smart Materials and Systems (CM@S) D-42119 Wuppertal Germany

## Abstract

Correction for ‘Polyterrylenes: synthesis and regioregularity effect on p-type charge transport and deep-red light photodetection in OFETs’ by Chitrak Ghosh *et al.*, *Chem. Sci.*, 2026, **17**, 2703–2711, https://doi.org/10.1039/D5SC06452J.

The authors regret that the chemical structure of compound **2** was incorrect in the original paper.

For the reported compound **2**, the authors were not able to obtain a single crystal despite several attempts. However, during a follow-up work (shown below) using a shorter alkyl chain (*n*-butyl), and further reaction of the dibromo compound to generate the *p*-cyanobenzene derivative (**TER-PHCN**), a single crystal was obtained. The single crystal of **TER-PHCN** (CCDC # 2524128) showed that the bromines in **TER-C4-Br2** are located at different *peri*-positions compared to the structure that was originally assigned in the published paper for compound **2**. Based on this crystal structure, the authors realized that the published structure of compound **2** needs to be corrected.

Synthesis of **TER-PHCN** and its SCXRD structure:
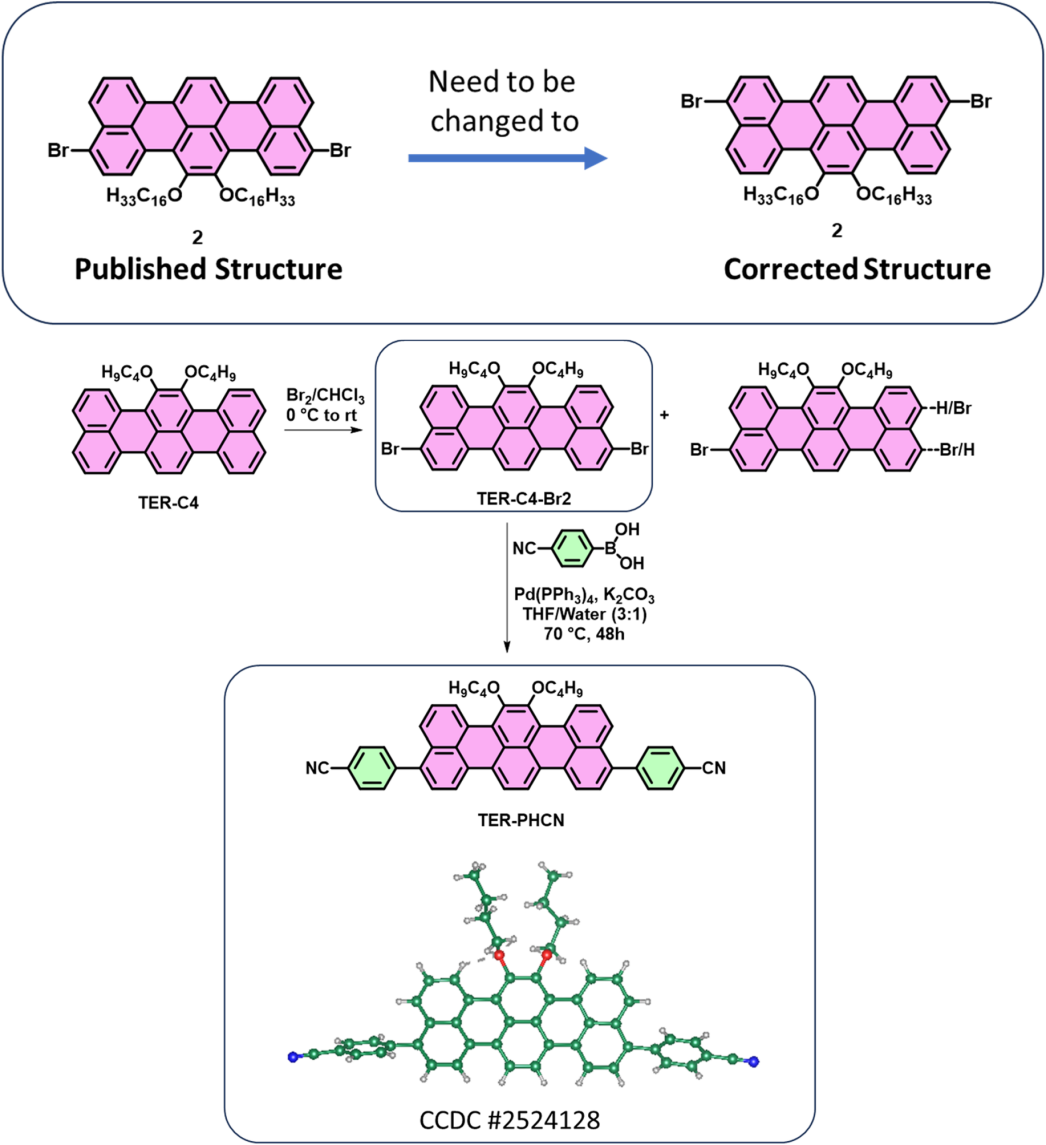


Both the published and corrected structures of compound **2** are symmetric and would produce identical NMR spectra. The published structure of compound **2** was the most probable based on literature reports on perylene-based systems (Ref: *Chem. Eur. J.* 2017, **23**, 9419–9424) and the electron-donating mesomeric effect of oxygen atoms. However, it is surprising and unexpected that the bromine atoms are located at the other *peri*-positions in **TER-C4-Br2**. Since the methods of bromination and isolation of the pure products were the same for both C4 and C16 chains, the authors believe that the published structure of compound **2** will adopt the location of bromine atoms similar to the **TER-C4-Br2**. Although the authors did not obtain the crystal structure for compound **2**, based on the crystal structure of **TER-PHCN** (CCDC # 2524128), the published structure of compound **2** can be changed to the corrected structure.

## Corrections to the main manuscript

Importantly, these changes in structure do not affect any spectral changes, observations, significance, direction, or the conclusions of the original article. All the compounds and their spectra, experimental data, and tables remain the same.

The figures where the structures of compound **2** and its polymer appeared need to be replaced with the corrected structure, as given here (Graphical abstract image, Fig. 1, Scheme 1, Fig. 3).

Graphical abstract image:
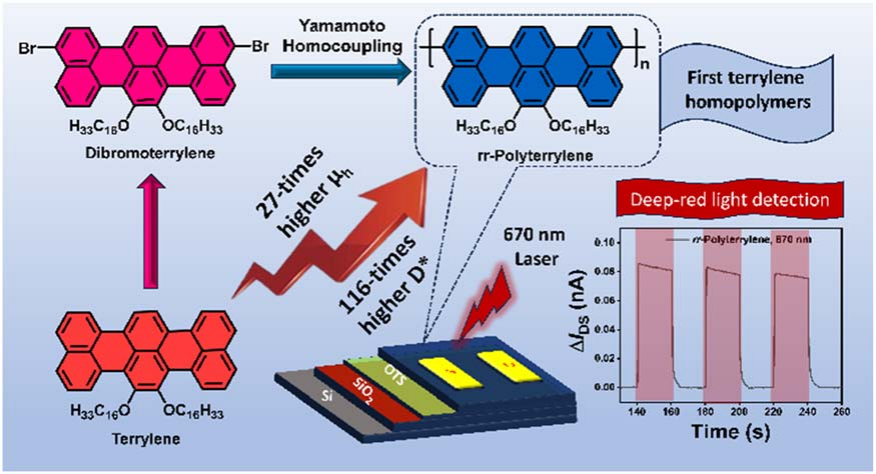


**Fig. 1:**
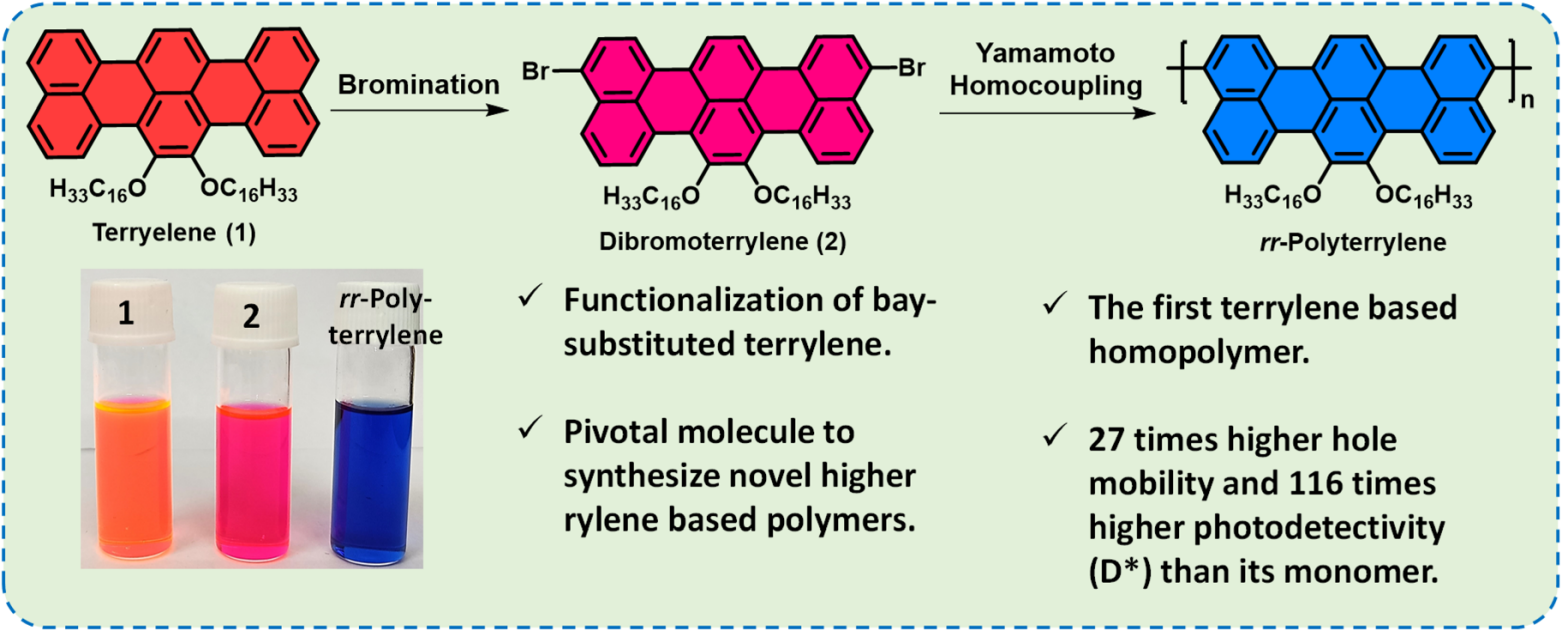
Schematic representation of functionalization of terrylene. Vial pictures showing the solutions of compounds **1**, **2** and ***rr*-Polyterrylene** in chloroform under daylight.

**Scheme 1:**
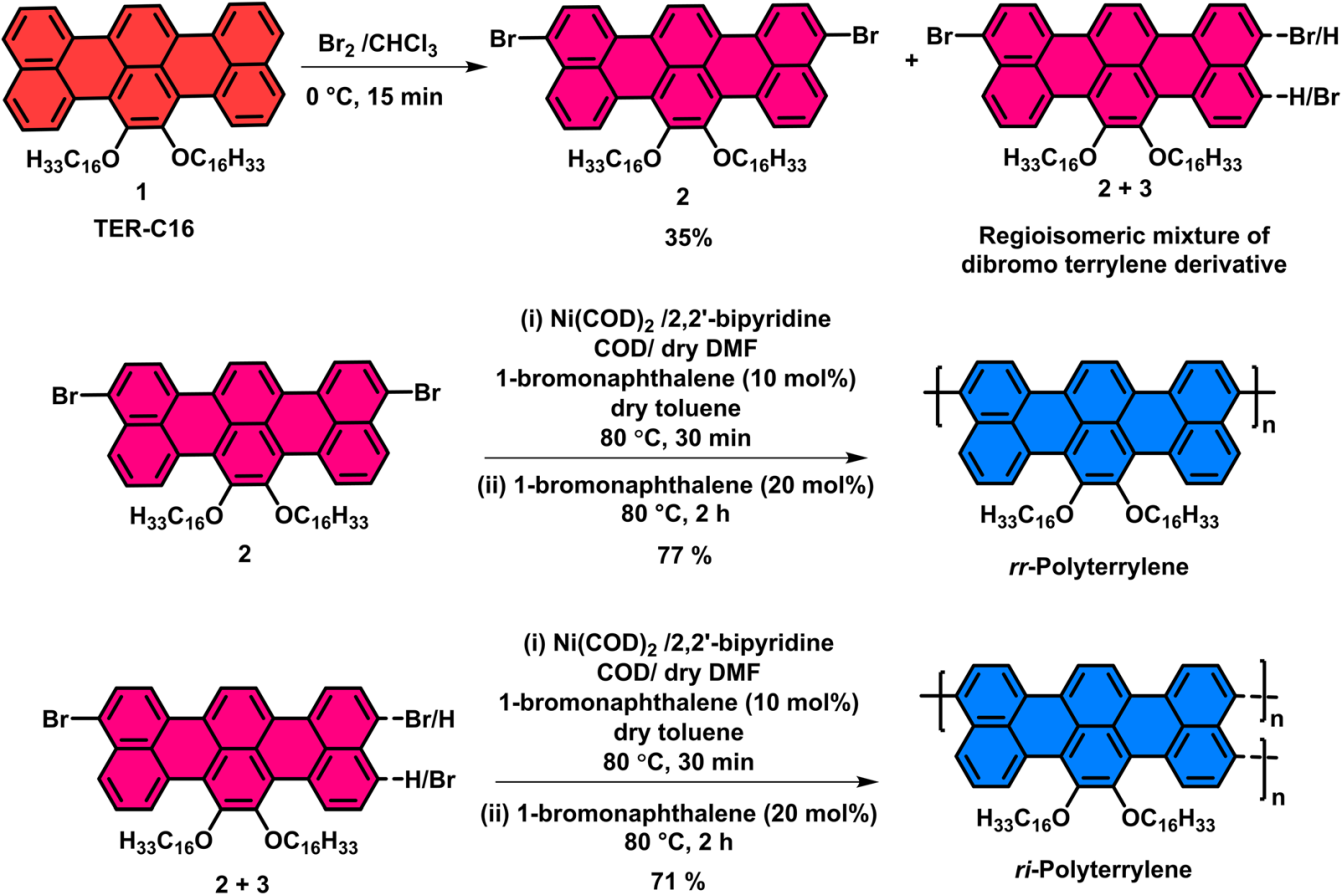
Synthesis of terrylene-based homopolymers, ***rr*-Polyterrylene** and ***ri*-Polyterrylene**.

**Fig. 2:**
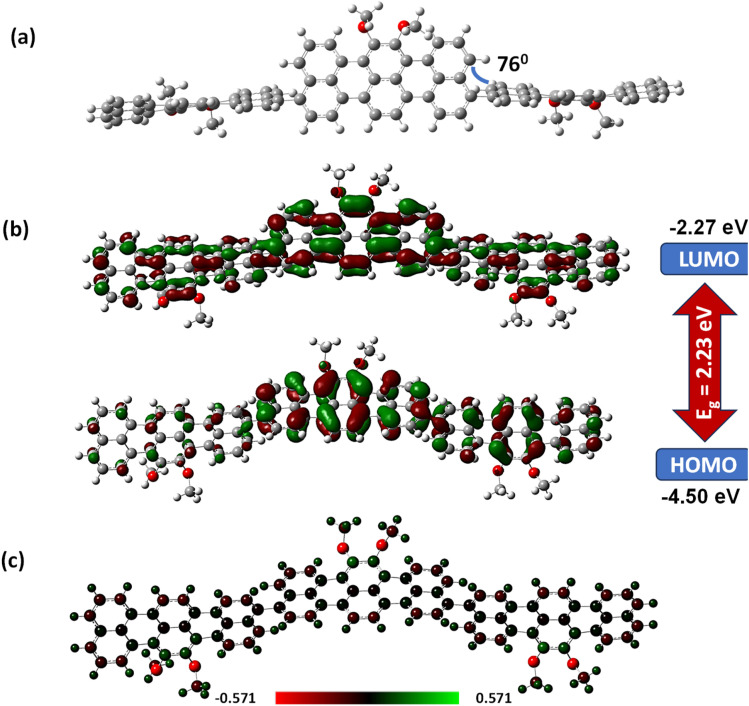
DFT calculations using the Gaussian16 program with the 6-31G basis set using the B3LYP method showing (a) optimized structure, (b) HOMO and LUMO distribution, and (c) charge distribution of ***rr*-Polyterrylene**.

In the ‘Theoretical studies’ section, two sentences need to be modified slightly:

‘The dihedral angle between two terrylene subunits in the *peri*-position is about 76° as the other two remaining hydrogen atoms at the *peri*-position of terrylene prevent polyterrylene from achieving a fully planar polymer configuration (Fig. 3a).’

‘In context with DFT calculations, polyterrylene showed a slightly uplifted HOMO and low-lying LUMO energy levels compared to 7,8-bis(methyloxy)terrylene, delineating a modest reduction of the band gap of 0.14 eV, which correlates well with their experimental bandgap differences.’

Additional corrections and the new crystal structure data have been added to an updated version of the SI (Fig. S3 and S10, Table S2).

The Royal Society of Chemistry apologises for these errors and any consequent inconvenience to authors and readers.

## Supplementary Material

SC-017-D6SC90068B-s001

SC-017-D6SC90068B-s002

