# Supplementary material for: Correction: Polyterrylenes: synthesis and regioregularity effect on p-type charge transport and deep-red light photodetection in OFETs
Source: Chem Sci. 2026 Mar 24;17(16):8300–4. doi: 10.1039/d6sc90068b (PMC13011975; doi:10.1039/d6sc90068b)
Supplement: SC-017-D6SC90068B-s001 [file SC-017-D6SC90068B-s001.pdf]

Supplementary Information (SI) for Chemical Science.
This journal is © The Royal Society of Chemistry 2026

Electronic Supplementary Information (ESI†)

Polyterrylenes: Synthesis and Effect of Regioregularity on p-Type Charge Transport and Deep-red Light Photodetection in OFETs

Chitrak Ghosh, # [a] Minji Chung, # [b] Hayeong Park,[b] Aniket Jitendra Talreja,[a] Ullrich Scherf,[c] Joon Hak Oh[b]* and Suman Kalyan Samanta[a]*

[a]Department of Chemistry, Indian Institute of Technology Kharagpur, Kharagpur 721302, India.

[b]School of Chemical and Biological Engineering, Institute of Chemical Processes, Seoul National University, Seoul 08826, Republic of Korea.

[c]Macromolecular Chemistry Group (buwmakro) and Wuppertal Center for Smart Materials and Systems (CM@S), D-42119 Wuppertal, Germany

Experimental Section.

Materials and methods: All the reagents, starting materials (Br_2, $Ni(COD)_2$, COD, 1-bromonaphthalene, and 2,2'-bipyridine), solvents, and inorganic salts were purchased from commercial suppliers and were used without further purification. Solvents were dried as per the literature procedure prior to use according to the requirements. Thin-layer chromatography (TLC) on silica gel GF_{254} was used to determine R_f values, and visualization was performed under UV light at 254 nm. Column chromatography was performed on Merck silica gel (100-200 mesh) with the eluent as mentioned. ^1H (400 MHz), ^1H (500 MHz), and ^{13}C (125 MHz) NMR spectra were recorded in a Bruker 400 UltraShield and Bruker Ascend-500 NMR spectrometer in deuterated solvents at ambient temperature (300 K). Chemical shifts are reported in ppm (δ) relative to tetramethylsilane (TMS) as the internal standard ($CDCl_3$ δ 7.26 ppm for ^1H and 77.0 ppm for ^{13}C). Single-crystal data were collected on a Bruker D8-Venture X-ray diffractometer. Matrix-assisted laser desorption/ionization mass spectrometry was recorded in an Autoflex Speed LRF (Bruker) instrument. Thermogravimetric Analysis (TGA) was performed on a Pyris Diamond TG DTA (Perkin Elmer) instrument under argon with a 12 °C/min heating rate. Differential scanning calorimetry was performed in a DSC25 (TA Instruments) at a heating rate of 10 °C in both exothermic and endothermic scans.

Measurements of Optical Properties: UV-visible absorption spectra were recorded on a Shimadzu UV-2550 UV-vis spectrophotometer. All three compounds were taken as a solution in $CHCl_3$ with a 10 µM concentration. Optical band gaps were measured from the equation: $E_g^{opt} = 1240/\lambda_{onset}$.

Cyclic Voltammetry Experiments: The electrochemical properties were characterized by a three-electrode cell with a polished 2 mm glassy carbon as the working electrode, Pt as the counter electrode, and Ag/AgCl as the reference electrode. The electrolytic solution employed was 0.1 (M) tetrabutylammonium hexafluorophosphate in dry dichloromethane at a scan rate of 0.05 V/s under an argon atmosphere. The reference electrode was calibrated using a ferrocene/ferrocenium redox couple as an external standard. The LUMO and HOMO energy levels of the compounds were determined by using the empirical equation,[1]

$$E_{HOMO} = -5.1 - [E_{ox} - E_{1/2}(Fc/Fc^+)]\ eV$$

$$E_{LUMO} = E_{HOMO} + E_{g,opt}$$

Theoretical Calculations: The ground states of the molecules were optimized using the DFT/B3LYP method[2,3] with the 6-31G basis set in Gaussian16.[4]

Device fabrication methods: Field-effect transistors (FETs) with a bottom-gate, top-contact (BGTC) configuration were prepared to characterize the electrical performance of ***rr*-Polyterrylene** and ***ri*-Polyterrylene**. A highly *n*-doped (100) Si wafer (< 0.004 Ω·cm) with a thermally grown SiO_2 (300 nm, C_i = 11.5 nF cm^{-2}) was utilized as the substrate and gate dielectric. After piranha cleaning for 30 minutes and UV-ozone treatment for 20 minutes, the wafers were functionalized with *n*-octadecyltrimethoxysilane (OTS) to form a self-assembled

monolayer (SAM), following a reported method. In order to fabricate the thin films, the polymers were dissolved in anhydrous chloroform (5 mg mL^{-1}) and spin-coated onto the substrates at 1000 rpm for 30 s, followed by thermal annealing under a N_2 atmosphere at an optimized temperature for 10 min. Au contacts (40 nm) were thermally evaporated through a shadow mask to form source and drain electrodes with a channel length (L) of 50 μm and a width (W) of 1000 μm. The electrical performance of FETs was tested in N_2 atmosphere using a Keithley 4200 semiconductor parametric analyzer. The field effect mobility in the saturation regime was calculated using the following equation:

$$I_{DS,sat} = \frac{1}{2}\mu C_{\mathrm{i}}\frac{W}{L}(V_{\mathrm{G}} - V_{\mathrm{T}})^2$$

where I_{DS} is the drain-to-source current, μ is the mobility, and V_{G} and V_{T} are the gate voltage and threshold voltage, respectively.

Synthesis

Synthesis of 3,12-dibromo-7,8-bis(hexadecyloxy)terrylene (2):

A solution of 7,8-bis(hexadecyloxy)terrylene (**TER-C16)** (0.822 mmol) in 100 mL chloroform was stirred at 0 °C. A solution of bromine (1.64 mmol) in 10 mL of chloroform was added dropwise, and then the mixture was stirred at 0 °C for 15 min. The reaction mixture was brought to room temperature, and a saturated sodium bicarbonate solution was added. It was extracted with chloroform and dried over $MgSO_4$. Repetitive column chromatography with hexane on silica gel was performed to purify compound **2**. Another regioisomeric dibromo derivative of 7,8-bis(hexadecyloxy)terrylene was also formed, which could not be isolated by column chromatography.

For **2**, Isolated yield 35%, ^{1}H NMR (500 MHz, $CDCl_3$) δ 9.21 (d, J = 7.8 Hz, 2H), 8.06 (d, J = 8.3 Hz, 2H), 7.84 (s, 2H), 7.72 (d, J = 8.1 Hz, 2H), 7.63 (d, J = 8.1 Hz, 2H), 7.55 (t, J = 8.0 Hz, 2H), 3.97 (t, 4H), 1.92 – 1.84 (m, 4H), 1.52 – 1.44 (m, 4H), 1.27 (s, 48H), 0.88 (t, 6H). ^{13}C NMR (125 MHz, $CDCl_3$) δ 152.62, 132.62, 130.95, 130.56, 130.16, 129.83, 128.08, 127.43, 126.89, 123.34, 122.55, 121.16, 120.35, 73.23, 32.10, 30.83, 29.90, 29.84, 29.76, 29.53, 26.42, 22.85, 14.25. MALDI-ToF: calculated, 1014.4348; obtained, 1015.5690.

General synthesis procedure of polyterrylenes:

A 50 mL Schlenk flask was dried in an oven and cooled. The flask was then charged with $Ni(COD)_2$ (0.155 mmol) and 2,2'-bipyridine (0.155 mmol) under an inert atmosphere in a glove box. The Schlenk flask was then subjected to argon-vacuum cycles to ensure thorough deoxygenation. After that, dry, degassed DMF (1 mL) was injected, followed by the addition of deoxygenated COD (0.02 mmol). The reaction was then heated to 80 °C for 5 minutes, and then dibromoterrylene (0.062 mmol) and 1-bromonaphthalene (0.006 mmol) were added via syringe along with degassed toluene (4 mL). The reaction was stirred for 0.5 hour at 80 °C. Then, 1-bromonaphthalene (0.012 mmol) was added again via syringe and stirred for another 2 hours. It was then cooled to room temperature and quenched with 6 N HCl. After that, the organic layer was washed with water and brine. It was dried over Na_2SO_4, and the solvent was evaporated. It was then precipitated in cold methanol and washed with methanol, acetone, ethyl acetate, and hexane in Soxhlet conditions for one day with each solvent. Finally, the polymer was extracted with chloroform, then precipitated into cold methanol to yield a deep blue solid powder.

For ***rr*-Polyterrylene**: Yield obtained 77%; ^{1}H NMR (500 MHz, $CDCl_3$) δ 9.21, 8.36, 8.00, 7.58, 7.47, 4.14, 1.24, 0.84. M_w (kg/mol) = 177.15; M_w/M_n = 2.6; UV ($CHCl_3$), λ_{max}: 607 nm, T_d (5% weight loss): 316 °C.

For ***ri*-Polyterrylene**: Yield obtained 71%; ^{1}H NMR (500 MHz, $CDCl_3$) δ 9.26, 8.33, 7.97, 7.62, 7.47, 4.15, 1.26, 0.85. M_w (kg/mol) = 122.32; M_w/M_n = 2.6; UV ($CHCl_3$), λ_{max}: 604 nm, T_d (5% weight loss): 304 °C.

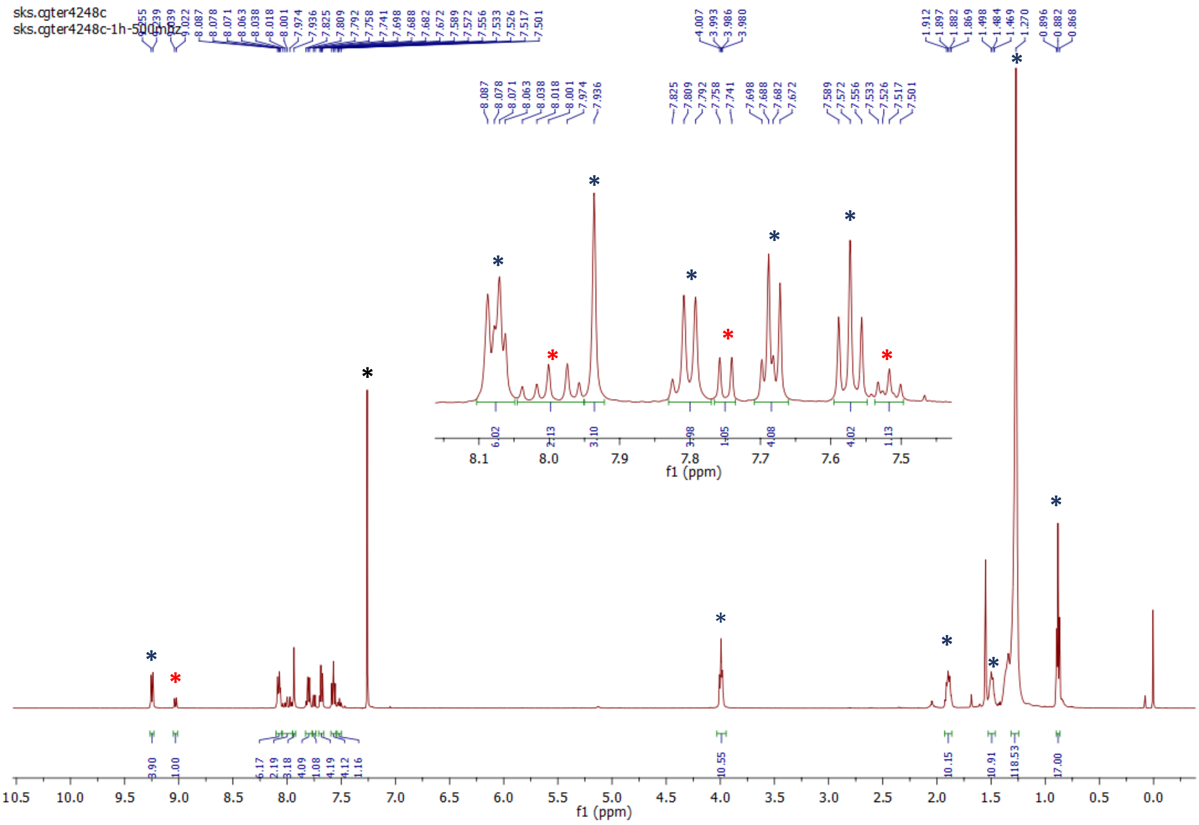

Fig. S1 ^{1}H NMR spectrum of dibromo-(hexadecyloxy)terrylene (**2+3**) in $CDCl_3$(*) at 298K. (* indicates peaks from **2+3**, * indicates peaks only from regioisomer **3**).

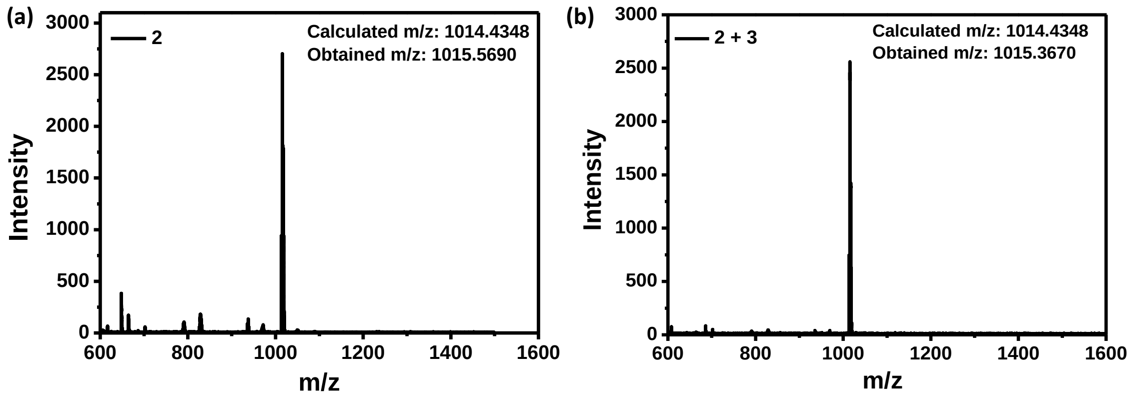

Fig. S2 MALDI-ToF spectra of (a) pure compound **2** and (b) compound **2 + 3**.

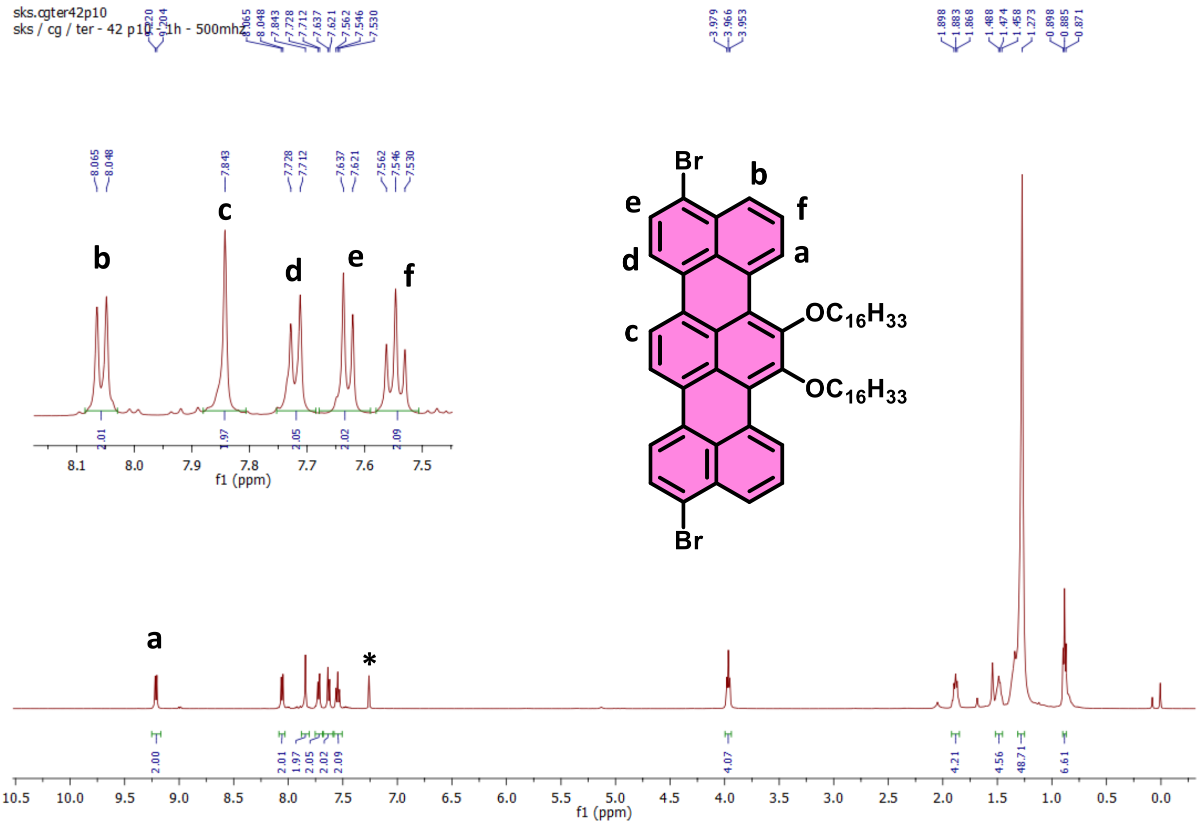

Fig. S3 ^{1}H NMR spectrum of **2** in $CDCl_3$(*) at 298K.

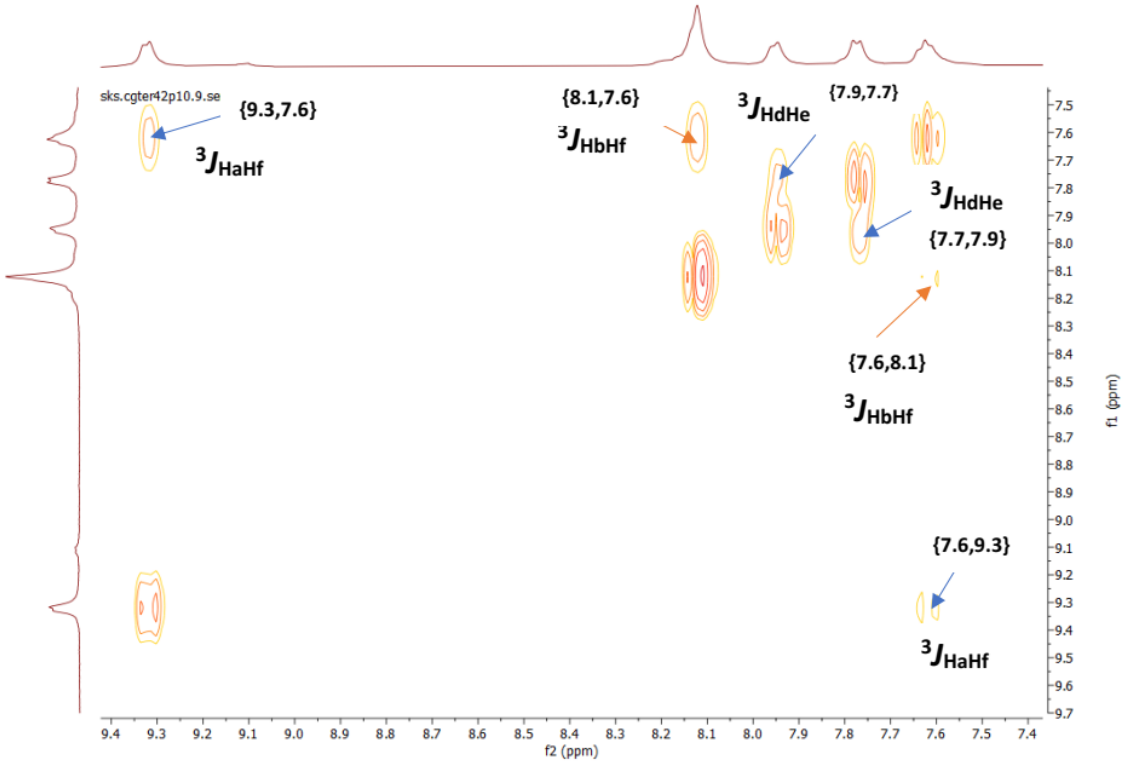

Fig. S4 2D COSEY NMR spectrum of **2** in $CDCl_3$ at 298K.

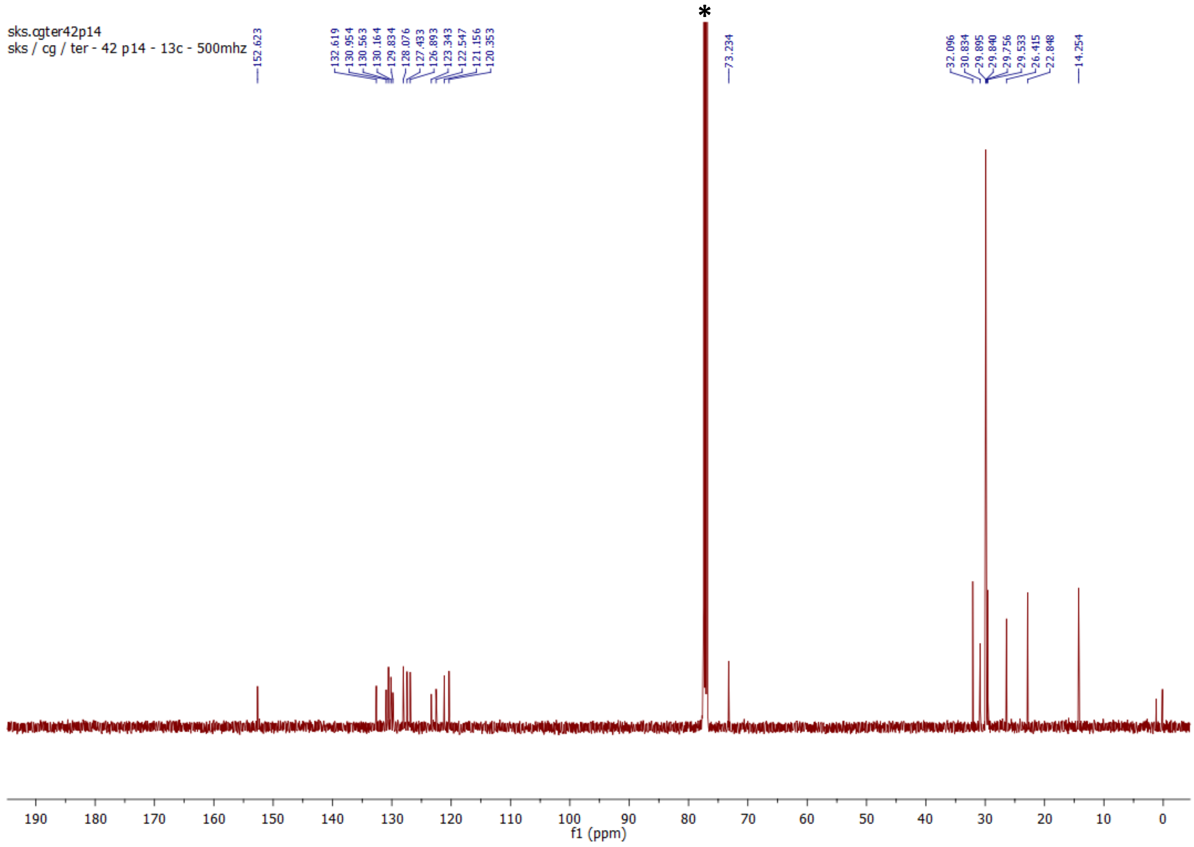

Fig. S5 ^{13}C NMR spectrum of **2** in $CDCl_3$ at 298K.

Table S1. Table for the optimized synthesis of soluble polyterrylenes.

Monomer	Amount of monomer	Time	Total Yield	Well Soluble Amount	M_n (kg mol^{-1})	M_w (kg mol^{-1})	PDI
**2**	63 mg	24 h	43 mg	8 mg	335.69	740.45	2.20
**2**	67 mg	1 h	44 mg	6 mg	294.38	437.06	1.75
**2**	70 mg	30 min	46 mg	9 mg	193.38	344.05	1.77
**2** + 0.1 eqv. 1-bromonapthalene	375 mg	30 min	243 mg	243 mg	66.13	177.15	2.67

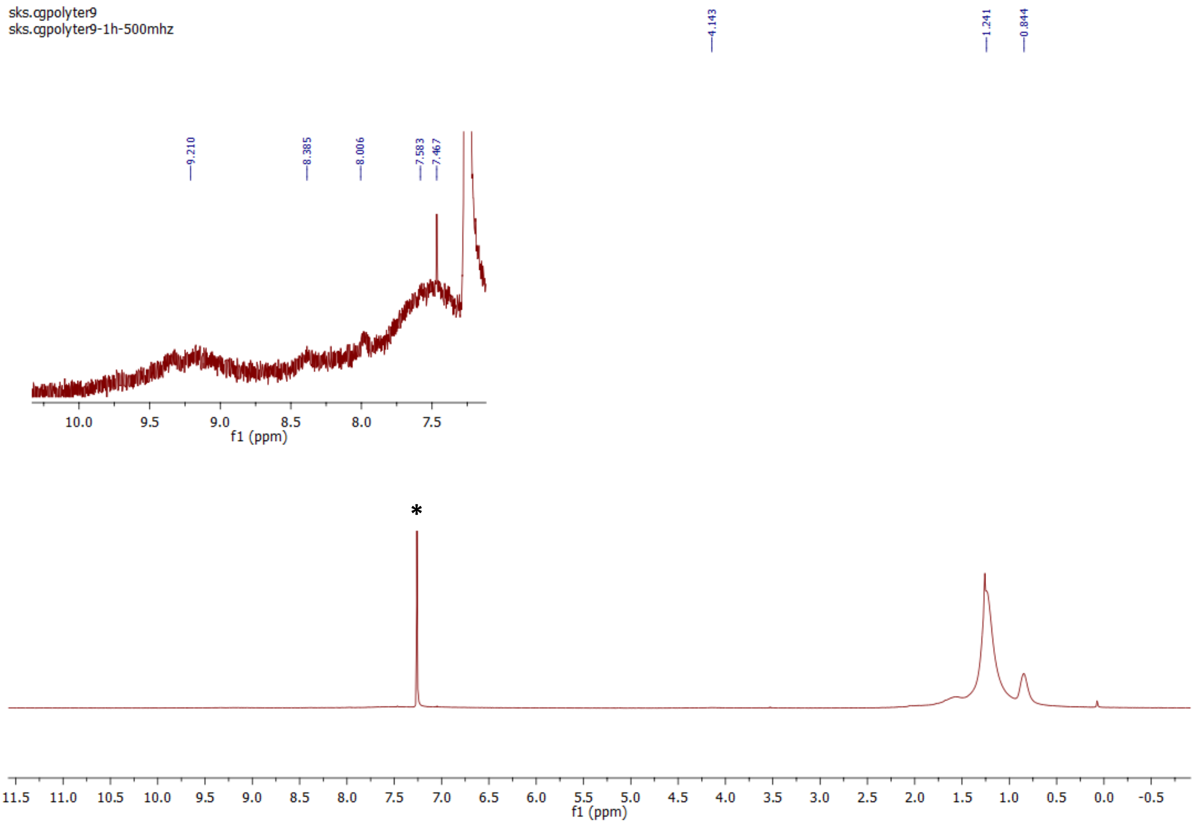

Fig. S6 ^{1}H NMR spectrum of ***rr*-polyterrylene** in $CDCl_3$(*) at 298K.

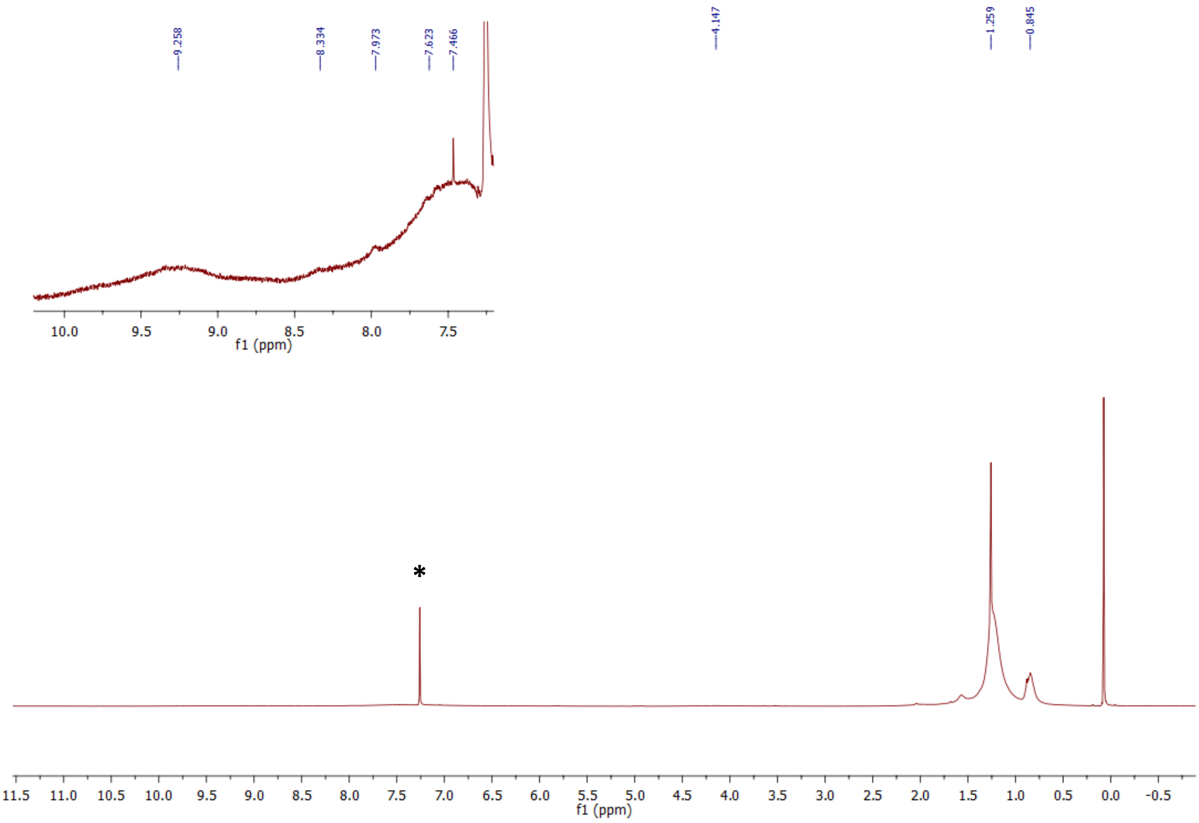

Fig. S7 ^{1}H NMR spectrum of ***ri*-polyterrylene** in $CDCl_3$(*) at 298K.

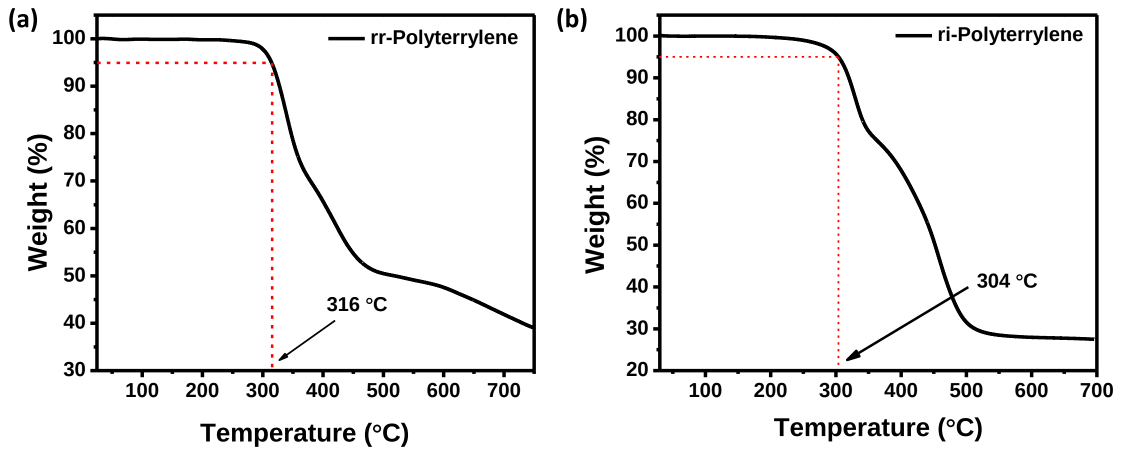

Fig. S8 Thermogravimetric analysis thermograms of (a) ***rr*-Polyterrylene** and (b) ***ri*-Polyterrylene**.

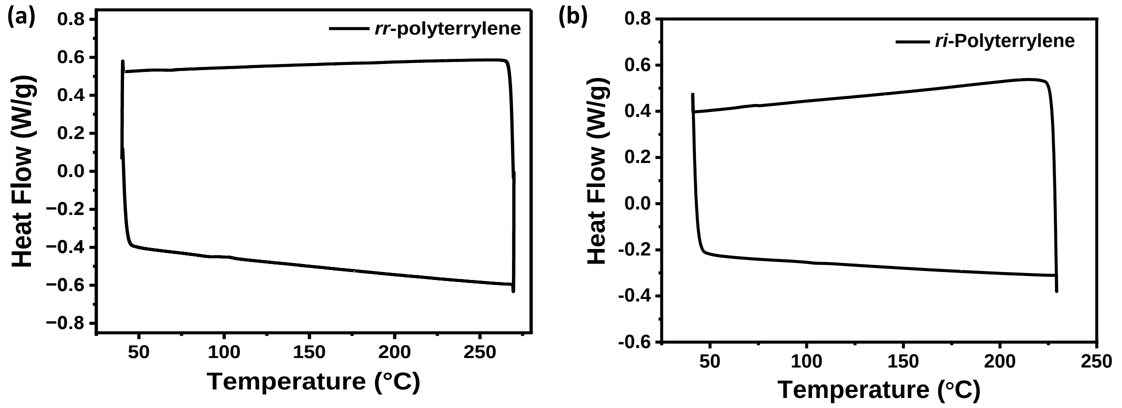

Fig. S9 Differential scanning calorimetry (DSC) thermograms of (a) ***rr*-Polyterrylene** and (b) ***ri*-Polyterrylene**.

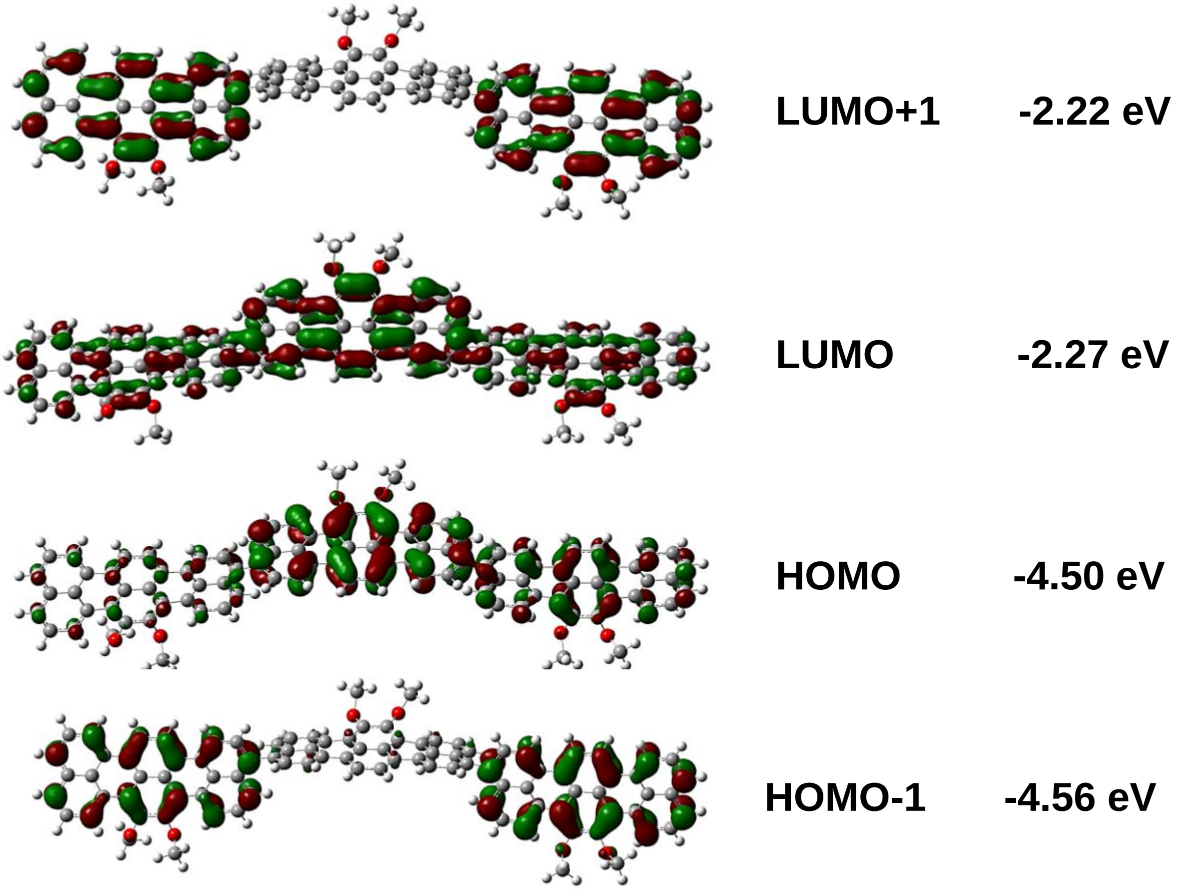

Fig. S10 DFT calculated energy levels and distributions of HOMO-1, HOMO, LUMO and LUMO+1 for ***rr*-Polyterrylene**.

Table S2. Cartesian coordinates of the DFT optimized structure of ***rr*-Polyterrylene.**

Energy = -4142.419724 Hartree

C -3.64813800 -0.49343600 -2.07967300

C -2.91387000 -0.99467100 -1.00401900

C -1.44603800 -0.96650100 -0.98038500

C -0.71682600 -0.26382200 -1.94252000

C -3.62111200 -1.52592000 0.12161900

C -0.73010800 -1.66625500 0.04495700

C -1.45163700 -2.35624400 1.08136400

C -2.91076800 -2.15940600 1.20191000

C -0.70835300 -3.21871400 1.90517700

C 0.70762400 -3.26518200 1.84566000

C 1.44357600 -2.41389600 1.01351400

C 0.70864000 -1.68396400 0.01764300

C 1.41502900 -0.99142400 -1.01829600

C 0.67607100 -0.27315800 -1.96019200

C -5.05096200 -0.43366300 -2.05037100

C -5.76982900 -0.85350700 -0.93950000

C -5.05704300 -1.41866900 0.17142700

H -5.58353100 -0.02712000 -2.90413600

C -5.74392900 -1.88547400 1.32573300

C -5.03932500 -2.40906700 2.38891900

C -3.63932100 -2.54885700 2.33219700

H -5.56462600 -2.73383000 3.28160500

H -3.11783000 -2.97619200 3.17274200

H -3.14203900 -0.13478800 -2.96749000

C 2.89967100 -2.20256600 1.13210900

C 3.60177500 -1.55985700 0.05565200

C 3.62149000 -2.56773700 2.27358000

C 2.88318500 -1.03274200 -1.06516600

C 5.03509000 -1.43400900 0.11008100

C 5.01784000 -2.39962000 2.34192400

H 3.09635000 -2.99275900 3.11734600

C 3.61122000 -0.53325400 -2.14506200

C 5.74131100 -0.87015100 -1.00639600

C 5.72060000 -1.87835700 1.27444900

H 5.54297300 -2.70158900 3.24254300

C 5.01504100 -0.46700600 -2.11886000

H 3.10018400 -0.17642200 -3.03096900

H 5.54261400 -0.06197000 -2.97646400

C -7.26254000 -0.74658600 -0.95116400

C -7.95717100 0.26592200 -0.20666100

C -8.00055100 -1.61985400 -1.73768500

C -7.25028200 1.20022700 0.59869100

C -9.39404900 0.34934200 -0.27724800

C -9.40377800 -1.56853900 -1.77003800

H -7.48299700 -2.37365300 -2.32224600

C -7.93655800 2.15479000 1.31861500

C -10.08672700 1.40580100 0.41218400

C -10.12026800 -0.62712400 -1.03005600

H -9.92360900 -2.28592700 -2.39274300

C -9.33733200 2.26234900 1.22758700

H -7.39615300 2.84710000 1.95653700

H -9.84512000 3.02594100 1.79364400

C -11.58864300 -0.62177800 -0.98892800

C -12.29240400 0.48178400 -0.40396700

C -12.32780500 -1.67881400 -1.52140400

C -11.55083400 1.53753900 0.23943000

C -13.73249600 0.53007800 -0.48490700

C -13.71942900 -1.67566200 -1.51218200

C -12.29221600 2.63956300 0.67741800

C -14.45753700 1.69346100 -0.03651400

C -14.44834300 -0.60090000 -1.00131900

C -13.70037800 2.70781200 0.55778300

C -15.91817900 -0.62150400 -0.98120400

C -16.62377600 0.57745000 -0.64472600

C -16.64978600 -1.76894800 -1.29977800

C -15.92275900 1.76239400 -0.22991100

C -18.05894800 0.59333700 -0.73989300

C -18.05668400 -1.76009400 -1.34713700

H -16.13894700 -2.69945200 -1.51359400

C -16.66824300 2.93284300 -0.03816100

C -18.76351300 1.80203100 -0.49365300

C -18.75255900 -0.59624000 -1.08936400

H -18.58693300 -2.67308100 -1.59825900

C -18.07138600 2.95058200 -0.17481900

H -16.15467800 3.84415100 0.22110600

H -19.84655300 1.80415200 -0.57405600

H -19.83664300 -0.57150400 -1.14682000

H -18.60255300 3.88352300 -0.01399300

C 7.20430600 1.20248900 0.52738900

C 7.91803900 0.27221400 -0.27720100

C 7.88328500 2.16292400 1.24679700

C 7.23261200 -0.74762100 -1.02023900

C 9.35383200 0.36791600 -0.34887400

C 9.28307700 2.28389300 1.15330800

H 7.33745900 2.84964000 1.88612200

C 7.97767400 -1.61536200 -1.80705500

C 10.08828800 -0.60596600 -1.09804200

C 10.03715200 1.43526800 0.33407500

H 9.78399800 3.05496000 1.71506500

C 9.38038200 -1.55341000 -1.83869400

H 7.46595000 -2.37373000 -2.39092300

H 9.90757700 -2.26830000 -2.45836500

C 11.55555000 -0.59645300 -1.04189500

C 12.24539600 0.50673700 -0.44122300

C 12.30915500 -1.64973300 -1.56443100

C 11.49640800 1.58115100 0.15555500

C 13.68464200 0.53488000 -0.45495100

C 13.70198400 -1.62710300 -1.56417700

C 12.21341900 2.73867200 0.50293000

C 14.39063000 1.65595700 0.10341500

C 14.41726500 -0.54591700 -1.04655600

C 13.62932800 2.77932400 0.44464500

C 15.88707900 -0.50949500 -1.08481700

C 16.57122400 0.49941800 -0.33328000

C 16.64283700 -1.45223200 -1.78603200

C 15.84672900 1.55303100 0.31927300

C 18.00263400 0.44210700 -0.21594000

C 18.04997300 -1.46794900 -1.71874500

H 16.15123300 -2.19798800 -2.39869500

C 16.55032400 2.41441700 1.16923200

C 18.67071300 1.37309600 0.62486800

C 18.72133300 -0.55368800 -0.93120600

H 18.60048100 -2.21742100 -2.27826700

C 17.94827400 2.31704300 1.32593100

H 16.00877300 3.17227600 1.71762400

H 19.75144300 1.31710400 0.71526300

H 19.80354600 -0.58114400 -0.84666000

H 18.45270700 3.00520000 1.99683500

H 6.12414900 1.13855200 0.57543800

H 6.79926400 -1.79023700 1.31654700

H -6.82407800 -1.81340600 1.36157000

H -6.16972600 1.14401600 0.64621100

H -1.23269600 0.31992100 -2.69441500

H 1.18254900 0.30531200 -2.72276700

O 1.31451400 -4.18102200 2.72572900

O -1.37203200 -3.97582900 2.86762300

C -1.22763700 -5.43115400 2.90766000

H -1.09127400 -5.83790400 1.89970000

H -0.39045800 -5.71109900 3.54641800

H -2.16604700 -5.80171100 3.32447300

C 2.04336700 -5.29565900 2.09730300

H 1.37504800 -5.86391600 1.44233600

H 2.90497800 -4.93631700 1.53043700

H 2.37813900 -5.92008900 2.92582100

H 11.81308700 -2.52105100 -1.97304800

H 14.22713300 -2.48239000 -1.97062000

O 11.52327200 3.83888600 1.00596100

O 14.21052100 4.00727100 0.81380800

H -11.82219100 -2.54029100 -1.93859500

H -14.23320500 -2.53016800 -1.93347800

O -14.29909300 3.87950200 1.05123300

O -11.67875900 3.74354600 1.29380300

C -11.54683000 4.94600500 0.45037500

H -10.92752700 4.72534000 -0.42476200

H -12.53475400 5.29857200 0.14784200

H -11.05383600 5.68599200 1.08110300

C -14.41318100 3.96236600 2.51935900

H -14.89731500 4.91922800 2.71565100

H -15.03378200 3.14274800 2.89514400

H -13.42008300 3.93624400 2.97173900

C 11.64698300 5.15376600 0.37637100

H 12.47142400 5.71036300 0.82109500

H 11.79118300 5.05673600 -0.70521800

H 10.69801000 5.65644600 0.57213400

C 14.93200300 4.72568900 -0.25031300

H 15.25097600 5.66350700 0.20486400

H 15.80333400 4.16102300 -0.58913700

H 14.26336600 4.92402000 -1.09415100

Table S3. Hole mobilities during optimization of thermal annealing temperature.

	***ri*-Polyterrylene**			***rr*-Polyterrylene**		
**Annealing Temperature (°C)**	$\mu_{h,avg}$ **(cm^2 V^{-1} s^{-1})**	I_{on}/I_{off}	V_{th} **(V)**	$\mu_{h,avg}$ **(cm^2 V^{-1} s^{-1})**	I_{on}/I_{off}	V_{th} **(V)**
**150**	2.6×10^{-4}	5.8×10^{5}	-24.0	5.4×10^{-4}	2.1×10^{4}	-9.0
**180**	1.5×10^{-4}	8.1×10^{3}	-21.6	1.0×10^{-3}	1.3×10^{4}	-18.1
**210**	1.7×10^{-4}	2.3×10^{4}	-24.5	7.6×10^{-4}	1.9×10^{4}	-25.6

Table S4. Optoelectronic characteristics (P, R, EQE, D*) under 635 nm light condition. V_{ds} = -80 V.

**Compounds**	P_{MAX}	R_{MAX} **(A/W)**	**EQE$_{MAX}$ (%)**	D^*_{MAX} **(Jones)**
***rr*-Polyterrylene**	1.6 x 10^{4}	5.8 x 10^{-3}	1.10	2.0 x 10^{10}
***ri*-Polyterrylene**	1.1 x 10^{4}	6.2 x 10^{-4}	0.12	5.8 x 10^{9}
**TER-C16 (532 nm)**	1.6 x 10^{2}	7.2 x 10^{-4}	0.17	3.7 x 10^{8}

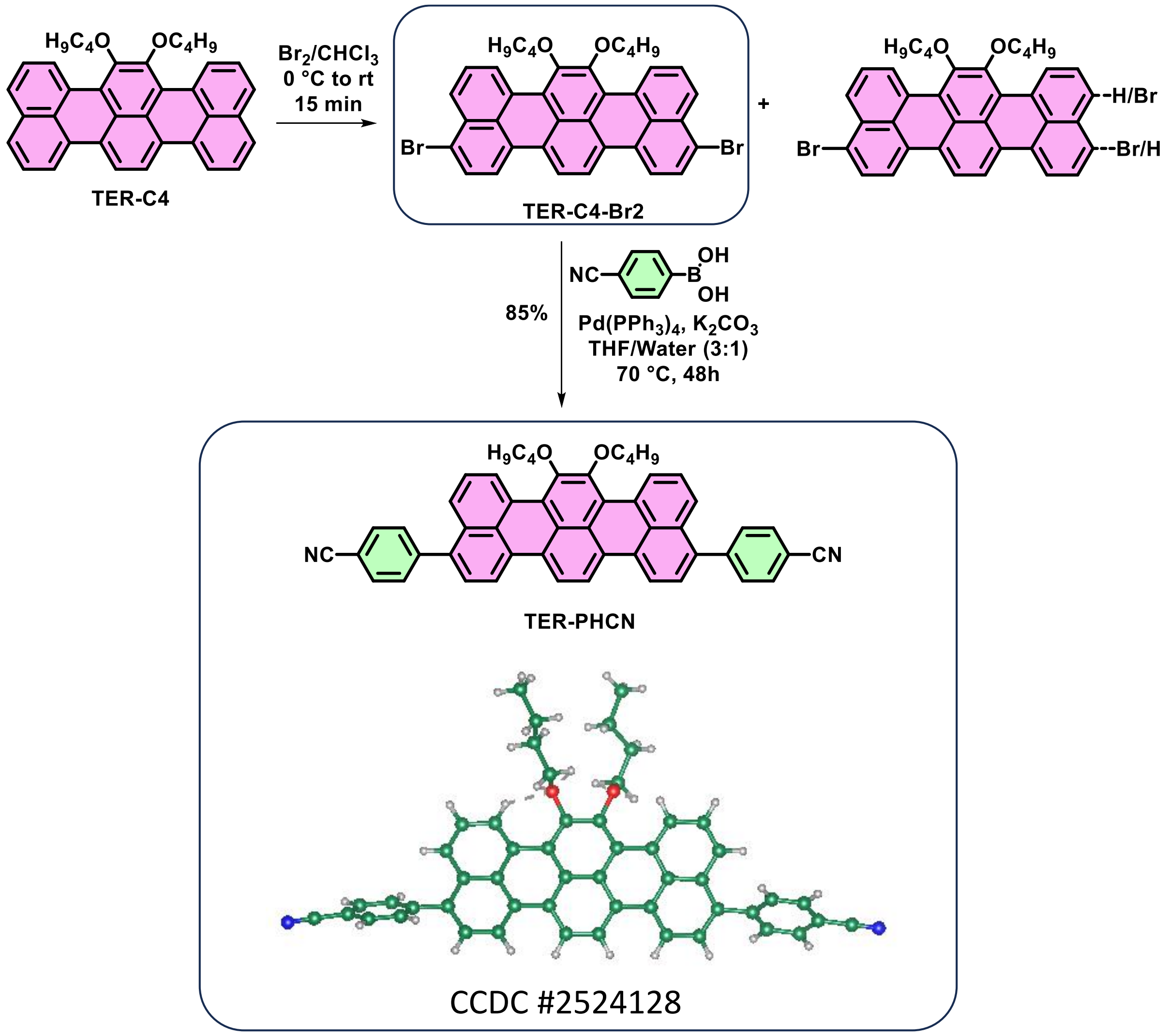

Scheme S1. Synthesis of **TER-PHCN**.

Synthesis:

7,8-bis(butyloxy)terrylene (**TER-C4**) was synthesized following our previously reported procedure.[5]

Synthesis of 3,12-dibromo-7,8-bis(butyloxy)terrylene (TER-C4-Br2): A solution of **TER-C4** (0.288 mmol) in 100 mL of chloroform was stirred at 0 °C. A solution of bromine (0.576 mmol) in 10 mL of chloroform was added dropwise, and then the mixture was stirred at 0 °C for 15 min. The reaction mixture was brought to room temperature, and a saturated sodium bicarbonate solution was added. It was extracted with chloroform and dried over $MgSO_4$. A repetitive column with hexane in silica gel was used to purify the compound **TER-C4-Br2**. Isolated yield 28%, ^{1}H NMR (400 MHz, $CDCl_3$) δ 9.33 (d, J = 8.5 Hz, 2H), 8.15 – 8.07 (m, 4H), 7.96 (d, J = 8.6 Hz, 2H), 7.78 (d, J = 8.8 Hz, 2H), 7.64 (t, J = 8.4 Hz, 2H), 4.07 (t, 4H), 1.91 (dd, 4H), 1.58 (d, 4H), 1.02 (t, 6H). MALDI-ToF: calculated, 678.0952; obtained, 678.7983.

Synthesis of 3,12-dibenzonitrile-7,8-bis(alkyloxy)terrylene (TER-PHCN): Compound **TER-C4-Br2** (30 mg, 0.044 mmol), (4-cyanophenyl)boronic acid (19 mg, 0.13 mmol), K_2CO_3 (62 mg, 0.44 mmol) and $Pd(PPh_3)_4$ (0.0044 mol, 5 mg) were taken in a 50 ml two-necked round-bottom flask and kept in argon. 6 ml of degassed tetrahydrofuran and 2 ml of degassed water were added to the two-necked round-bottom flask and again degassed for 15 minutes. After that, it was refluxed for 48 hours. Then the reaction mixture was cooled down to room temperature, and the organic layer was washed with water and brine and dried over Na_2SO_4. The obtained compound was purified through column chromatography to get compound **TER-PHCN** as a red solid (27 mg in total). Yield 85%. ^{1}H NMR (400 MHz, $CDCl_3$) δ 9.37 (d, J = 7.5 Hz, 2H), 8.31 (s, 2H), 8.28 (d, J = 7.9 Hz, 2H), 7.82 (d, J = 8.2 Hz, 4H), 7.69 (t, J = 7.8 Hz, 6H), 7.53 (t, J = 8.1 Hz, 2H), 7.46 (d, J = 7.8 Hz, 2H), 4.12 (t, 4H), 1.99 – 1.90 (m, 4H), 1.60 (dd, 4H), 1.02 (t, 6H). MALDI-ToF: calculated, 722.2933; obtained, 722.9396.

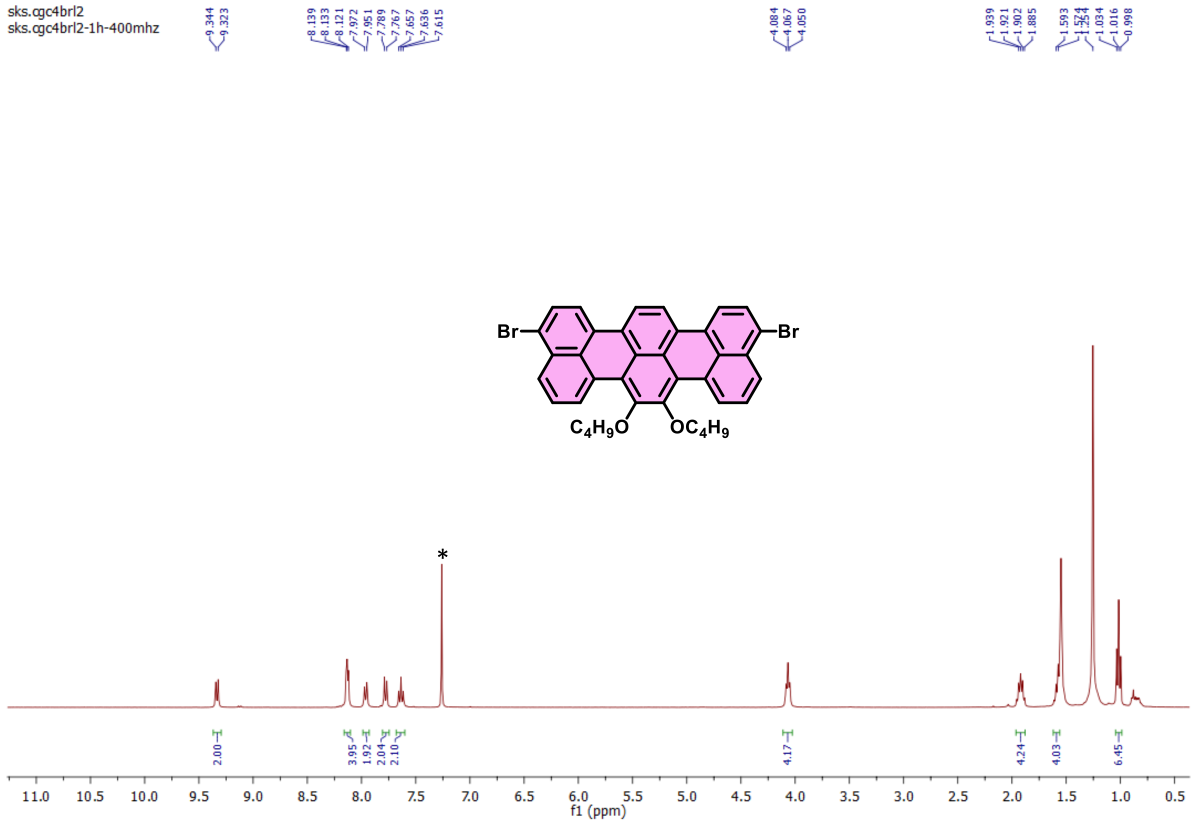

Fig. S11 ^{1}H NMR spectra of dibromo derivatives of **TER-C4-Br2** in $CDCl_3$(*) at 298K.

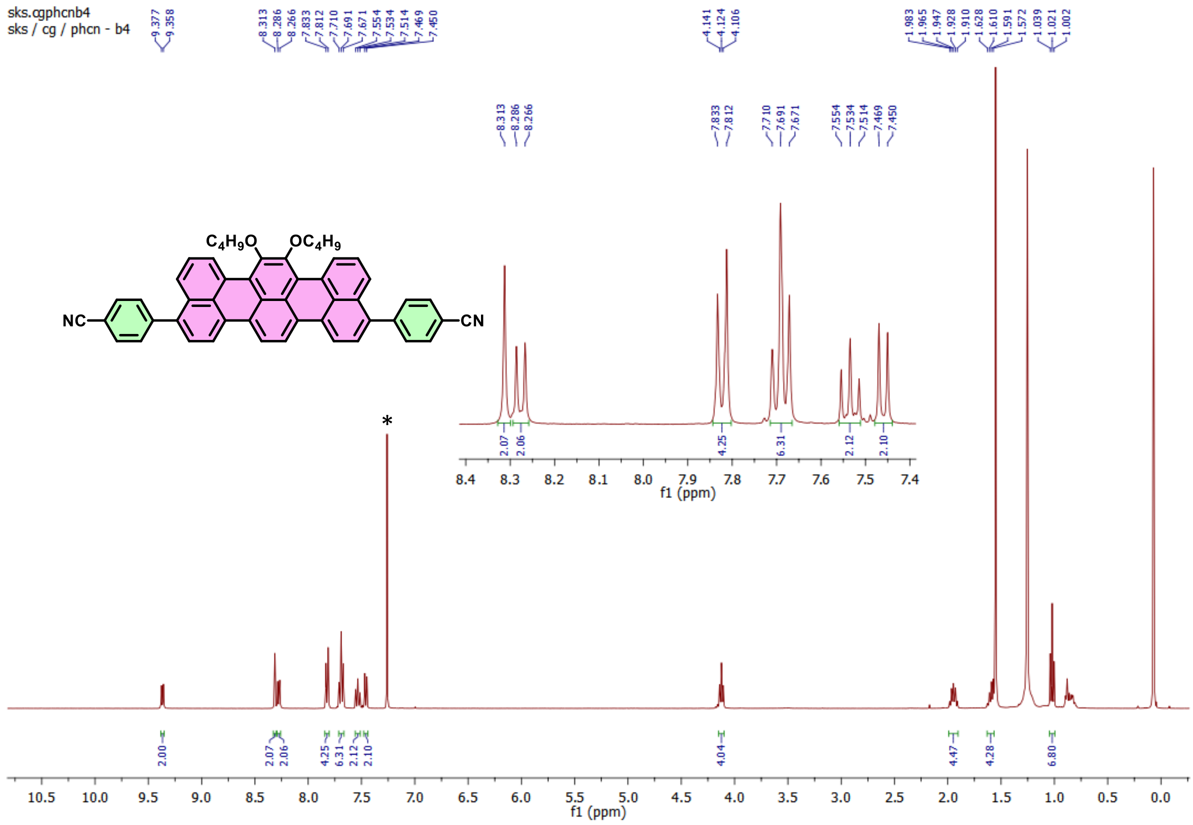

Fig. S12 ^{1}H NMR spectra of dibromo derivatives of **TER-PHCN** in $CDCl_3$(*) at 298K.

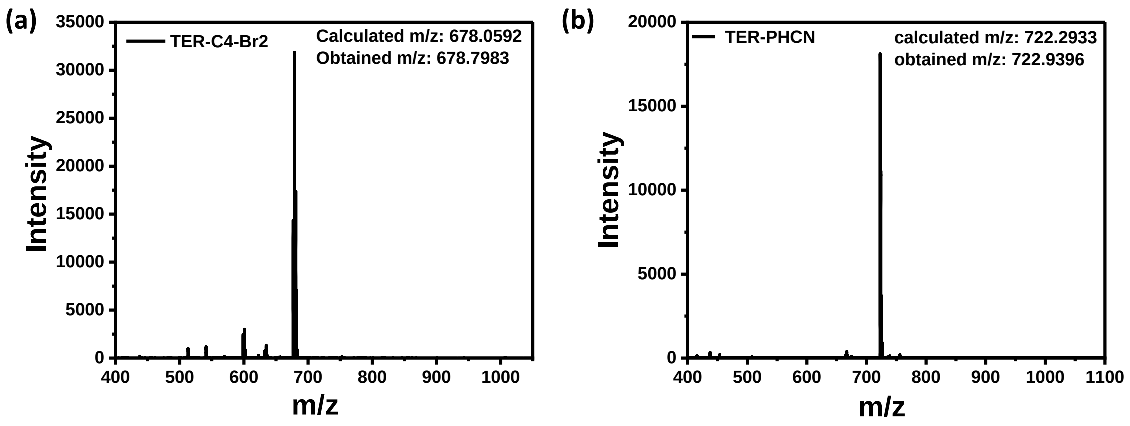

Fig. S13 MALDI-ToF spectra of (a) **TER-C4-Br2** and (b) **TER-PHCN**.

Table S5. Crystal data and structure refinement for **TER-PHCN**.

Identification code	SKS_CG_TER_C4PHCNR_0m_a
Empirical formula	$C_{52}H_{38}N_2O_2$
Formula weight	795.044
Temperature/K	120.00
Crystal system	triclinic
Space group	P-1
a/Å	8.3962(7)
b/Å	13.2577(13)
c/Å	18.6455(18)
α/°	80.895(3)
β/°	80.312(3)
γ/°	79.112(3)
Volume/Å^3	1991.9(3)
Z	2
ρ_{calc}g/cm^3	1.326
μ/mm^{-1}	0.079
F(000)	844.5
Crystal size/mm^3	0.4 × 0.3 × 0.3
Radiation	Mo Kα (λ = 0.71073)
2θ range for data collection/°	4.48 to 49.98
Index ranges	-10 ≤ h ≤ 10, -16 ≤ k ≤ 16, -22 ≤ l ≤ 22
Reflections collected	60934
Independent reflections	6990 [R_{int} = 0.1694, R_{sigma} = 0.1053]
Data/restraints/parameters	6990/0/599
Goodness-of-fit on F^2	1.050
Final R indexes [I>=2σ (I)]	R_1 = 0.0563, wR_2 = 0.1252
Final R indexes [all data]	R_1 = 0.1246, wR_2 = 0.1706
Largest diff. peak/hole / e Å^{-3}	0.53/-0.45

References.

1. Wang, Y.; Liao, Q.; Chen, J.; Huang, W.; Zhuang, X.; Tang, Y.; Li, B.; Yao, X.; Feng, X.; Zhang, X.; Su, M.; He, Z.; Marks, T. J.; Facchetti, A.; Guo, X. Teaching an Old Anchoring Group New Tricks: Enabling Low-Cost, Eco-Friendly Hole-Transporting Materials for Efficient and Stable Perovskite Solar Cells. *J. Am. Chem. Soc.* **2020**, *142* (39), 16632−16643.

2. Becke, A. D., Density-functional thermochemistry. I. The effect of the exchange-only gradient correction. *J. Chem. Phys.* **1992,** *96* (3), 2155-2160.

3. Lee, C.; Yang, W.; Parr, R. G., Development of the Colle-Salvetti correlation-energy formula into a functional of the electron density. *Physical Review B* **1988,** *37* (2), 785-789.

4. Frisch, M. J.; Trucks, G. W.; Schlegel, H. B.; Scuseria, G. E.; Robb, M. A.; Cheeseman, J. R.; Scalmani, G.; Barone, V.; Petersson, G. A.; Nakatsuji, H.; Li, X.; Caricato, M.; Marenich, A. V.; Bloino, J.; Janesko, B. G.; Gomperts, R.; Mennucci, B.; Hratchian, H. P.; Ortiz, J. V.; Izmaylov, A. F.; Sonnenberg, J. L.; Williams; Ding, F.; Lipparini, F.; Egidi, F.; Goings, J.; Peng, B.; Petrone, A.; Henderson, T.; Ranasinghe, D.; Zakrzewski, V. G.; Gao, J.; Rega, N.; Zheng, G.; Liang, W.; Hada, M.; Ehara, M.; Toyota, K.; Fukuda, R.; Hasegawa, J.; Ishida, M.; Nakajima, T.; Honda, Y.; Kitao, O.; Nakai, H.; Vreven, T.; Throssell, K.; Montgomery Jr., J. A.; Peralta, J. E.; Ogliaro, F.; Bearpark, M. J.; Heyd, J. J.; Brothers, E. N.; Kudin, K. N.; Staroverov, V. N.; Keith, T. A.; Kobayashi, R.; Normand, J.; Raghavachari, K.; Rendell, A. P.; Burant, J. C.; Iyengar, S. S.; Tomasi, J.; Cossi, M.; Millam, J. M.; Klene, M.; Adamo, C.; Cammi, R.; Ochterski, J. W.; Martin, R. L.; Morokuma, K.; Farkas, O.; Foresman, J. B.; Fox, D. J. *Gaussian 16 Rev. C.01*, Wallingford, CT, 2016.

5. Ghosh, C.; Park, H.; Hazra, A.; Mondal, B.; Chung, M.; Scherf, U.; Oh, J. H.; Samanta, S. K. Low-cost synthesis of soluble bay-substituted terrylenes for p-type charge transport and efficient photodetection on field-effect transistors. *J. Mater. Chem. C* **2025**, *13*, 7518 – 7527.
